# Serum IL-33, a new marker predicting response to rituximab in rheumatoid arthritis

**DOI:** 10.1186/s13075-016-1190-z

**Published:** 2016-12-13

**Authors:** Jérémie Sellam, Elodie Rivière, Alice Courties, Paul-Olivier Rouzaire, Barbara Tolusso, Edward M. Vital, Paul Emery, Gianfranco Ferraciolli, Martin Soubrier, Bineta Ly, Houria Hendel Chavez, Yassine Taoufik, Maxime Dougados, Xavier Mariette

**Affiliations:** 1 Université Paris 06, AP-HP St-Antoine hospital, Rheumatology Department, INSERM UMRS_938, DHU i2B, Paris, France; 2 Université Paris-Sud, AP-HP Hôpitaux Universitaires Paris-Sud, Rheumatology Department, Center for Immunology of Viral Infections and Autoimmune Diseases INSERM U1184, Le Kremlin Bicêtre, France; 3 Biological Immunology Department, ERTICa Research Group, Clermont-Ferrand University Hospital, Clermont-Ferrand, EA4677 France; 4 Rheumatology Department, Catholic University of the Sacred Heart, Roma, Italy; 5 NIHR Leeds Musculoskeletal Biomedical Research Unit, Leeds Teaching Hospitals NHS Trust, Leeds, UK and Leeds Institute of Rheumatic and Musculoskeletal Medicine, University of Leeds, Leeds, UK; 6 Rheumatology Department, Clermont-Ferrand University Hospital, Clermont-Ferrand, France; 7 AP-HP Bicêtre Hospital, Biological Immunology Department, INSERM U1184, Le Kremlin Bicêtre, France; 8 Department of Rheumatology - Hôpital Cochin, Paris Descartes University, Assistance Publique - Hôpitaux de Paris, INSERM (U1153), Clinical Epidemiology and Biostatistics, PRES Sorbonne Paris-Cité, Paris, France; 9 Service de Rhumatologie, Hôpital de Bicêtre, 78 rue du Général Leclerc, Le Kremlin Bicêtre, 94275 France; 10 Service de Rhumatologie, Hôpital Saint-Antoine, 184 rue du Faubourg Saint-Antoine, Paris, 75012 France

**Keywords:** Rheumatoid arthritis, Interleukin 33, Rituximab, B-cell, Personalized medicine

## Abstract

**Background:**

Recent works have suggested a possible link between interleukin (IL)-33 and B-cell biology. We aimed to study the possible association between serum IL-33 detection and response to rituximab (RTX) in rheumatoid arthritis (RA) patients in different cohorts with an accurate enzyme-linked immunosorbent assay (ELISA).

**Methods:**

Serum IL-33, rheumatoid factor (RF), anti-cyclic citrullinated peptide (anti-CCP), and high serum immunoglobulin (Ig)G levels were assessed in 111 RA patients receiving a first course of 2 g RTX (cohort 1) in an observational study and in 74 RA patients treated with the same schedule in routine care (cohort 2). Univariate and multivariate analyses identified factors associated with a European League Against Rheumatism (EULAR) response at 24 weeks.

**Results:**

At week 24, 84/111 (76%) and 54/74 (73%) patients reached EULAR response in cohorts 1 and 2, respectively. Serum IL-33 was detectable in only 33.5% of the patients. In the combined cohorts, the presence of RF or anti-CCP (odds ratio (OR) 3.27, 95% confidence interval (CI) 1.13–9.46; *p* = 0.03), high serum IgG (OR 2.32, 95% CI 1.01–5.33; *p* = 0.048), and detectable serum IL-33 (OR 2.40, 95% CI 1.01–5.72; *p* = 0.047) were all associated with RTX response in multivariate analysis. The combination of these three factors increased the likelihood of response to RTX. When serum IL-33 detection was added to seropositivity and serum IgG level, 100% of the patients with the three risk factors (corresponding to 9% of the population) responded to RTX (OR versus patients with none of the three risk factors 29.61, 95% CI 1.30–674.79; *p* = 0.034).

**Conclusion:**

Detectable serum IL-33 may predict clinical response to RTX independently of, and synergistically with, auto-antibodies and serum IgG level.

**Trial registration:**

NCT01126541; 18 May 2010.

**Electronic supplementary material:**

The online version of this article (doi:10.1186/s13075-016-1190-z) contains supplementary material, which is available to authorized users.

## Background

Interleukin (IL)-33 is one of the most recently discovered members of the IL-1 cytokine family mediating its biological effects via its binding to its receptor suppression of tumorigenicity (ST)2 [1]. IL-33 is upregulated in both resident cells and inflammatory infiltrating cells and is released in case of cell injury, thus acting as an alarmin [2, 3]. IL-33 induces production of Th2 cytokines and eosinophilia, and may activate mast cells that can in turn release several pro-inflammatory cytokines [4]. IL-33 investigations have been mainly devoted to asthma and allergy, with the development of a targeted IL-33/ST2 axis therapeutic strategy [5].

IL-33 may also be involved in rheumatoid arthritis (RA) pathogenesis. IL-33 administration exacerbates collagen-induced and K/BxN serum-mediated murine arthritis, and disease severity is reduced in mice treated with sST2-Fc fusion protein or anti-IL-33 monoclonal antibody [6–8]. Extracellular IL-33 is a critical enhancer of tumor necrosis factor (TNF)-induced RA synovial fibroblast activation [9] and could activate osteoclastogenesis [10, 11]. In patients with RA, biomarker studies have suggested that the serum level of IL-33 could reflect clinical activity [12] and disease severity, or predict carotid plaque progression [13]. However, the role of IL-33 could be paradoxical since, in K/BxN serum transfer-induced arthritis, ST2 but not IL-33 blockade may improve arthritis [14, 15]. Moreover, IL-33-stimulated mast cells could also suppress monocyte activation [16] and intracellular IL-33 also has anti-osteoclastogenic and anti-inflammatory properties [11].

Some recent works have suggested a possible link between IL-33 and B-cell biology [17]. In mice, IL-33 enhances immunoglobulin (Ig)M synthesis and markedly induces and activates B1 cells in an ST2-dependent manner [18]. Additionally, IL-33 could also induce regulatory B cells to produce IL-10, attenuating mucosal inflammation in the gut [19].

Using a transcriptomic approach, we have found that increased IL-33 mRNA expression in the whole blood of patients with RA was predictive of the response to rituximab (RTX), a targeted B cell-depleting agent [20]. We aimed to investigate, using an accurate and simple enzyme-linked immunosorbent assay (ELISA), the possible association between a detectable serum level of the IL-33 protein and a response to RTX in RA patients in different cohorts.

## Methods

### Patients

A total of 224 patients with RA for at least 6 months and fulfilling the American College of Rheumatology (ACR) 1987 criteria were included in the SMART study (NCT01126541). This study is a 2-year, national, multicenter, randomized open-label study evaluating the efficacy and tolerability of two doses of RTX for re-treatment after one initial course of RTX at a usual dose (1000 mg on days 1 and 15) described previously [21]. All patients had active disease, defined by a Disease Activity Score in 28 joints (DAS28) using C-reactive protein (CRP) (DAS28-CRP) >3.2, with ≥6/66 swollen and ≥6/68 tender joints, or a CRP ≥10 mg/L, or an erythrocyte sedimentation rate (ESR) ≥28 mm/h. Erosive status was based on the reading of hand and feet X-rays by the investigators in each center. Each patient received a stable dose of methotrexate (MTX) (≥10 mg/week for at least 4 weeks) and had experienced an inadequate response or intolerance to TNF inhibitors, or had contraindications to TNF inhibitors. All patients in the SMART study received one course of RTX (two 1000 mg infusions, given on days 1 and 15) with usual pre-medication (methylprednisolone, acetaminophen, and antihistamine). In this ancillary study, 111 patients from the 224 were included due to the availability of the serum samples for IL-33 assessment and were designated as cohort 1.

We also studied 32 RA patients from Leeds (UK) and 42 from Clermont-Ferrand (France) treated in real life with a first course of RTX using the same schedule. In order to match the numbers of patients in cohort 1, these two groups of patients from Leeds and Clermont-Ferrand were merged and called cohort 2. There were no overlaps between the two cohorts.

For all patients, treatment efficacy was evaluated 24 weeks after the first RTX infusion according to European League Against Rheumatism (EULAR) response [22]. Patients were then classified as responders (good or moderate) or non-responders after this first course of RTX.

### Assessment of serum IL-33, auto-antibodies, and serum IgG

We first used an ELISA IL-33 kit (DuoSet, R&D Systems) for protein assessment, and preliminary presented as an abstract an association between serum IL-33 level and RTX response in a single cohort [23]. However, this kit was not validated for human sera and additional experiments have confirmed the need for caution about the accuracy of this kit for sera measurements [24]. Recommendations from the manufacturer advise us to use another assay for human sera, named Quantikine and also provided by R&D Systems. We aimed to use this more accurate ELISA in order to investigate the possible association between a detectable serum level of IL-33 and a response to RTX in RA patients in the different cohorts.

Using a sample obtained prior to the initiation of RTX in the two cohorts, we measured the serum IL-33 level using the Quantikine kit, which is a solid-phase ELISA with a 6.25 pg/mL lower limit threshold according to the manufacturer’s instruction [24]. The assessment of rheumatoid factor (RF), anti-cyclic citrullinated peptide (anti-CCP) antibodies, and serum IgG level were also performed. The upper limit of normal (ULN) for serum IgG was 12.7 g/L and high serum IgG level was a value above this cut-off, as described previously [25].

### Statistical analysis

Continuous data are described as means and standard deviations (mean ± SD) or as medians and ranges (median (range)).

For all analyses, since serum IL-33 is not systematically detectable, we presented qualitative results as detectable serum IL-33 or undetectable serum IL-33. Among patients of the SMART cohort, we compared patients who were assessed for serum IL-33 level and those who were not due to the availability of the serum samples. Student and Wilcoxon tests were used to compare quantitative values and the Chi-squared test was used for qualitative values.

We analyzed by logistic regression the relationship between EULAR response at 24 weeks and the following four explanatory variables: 1) high disease activity defined as DAS28-CRP >5.1; 2) high serum IgG level; 3) auto-antibody status (i.e., the presence or absence of RF and/or anti-CCP antibodies), since all of them have been found previously as associated with subsequent RTX response [25, 26]; and 4) detectable serum IL-33. All these explanatory variables were entered in a stepwise multivariate model systematically adjusted on RF or anti-CCP positivy (entry level, *p* = 0.15; level for staying in the model, *p* = 0.10). Results are expressed as odds ratios (ORs) with 95% confidence intervals (CIs). A *p* value <0.05 was considered as significant. Statistical analysis was performed with SAS 9.4 software.

## Results

### Characteristics of the populations

The characteristics of patients from cohort 1 are presented in Table 1. There was no significant difference between the 111 patients that underwent IL-33 assessment and the others that participated in the main SMART trial (*n* = 113) who did not (Table 1). Available characteristics of patients from cohort 2 are presented in Additional file 1 (Table S1).

**Table 1:** Baseline characteristics of RA patients included in the IL-33 ancillary study from the SMART trial and a comparison between the patients in the SMART cohort included in the IL-33 study (cohort 1, *n* = 111) and the rest of the cohort not assessed for serum IL-33 level (*n* = 113)

	Cohort 1	Patients in the SMART cohort without IL-33 dosage	Whole SMART cohort	Inter-group comparison
	(*n* = 111)	(*n* = 113)	(*n* = 224)	
Age (years)	56 ± 11	56 ± 11	56 ± 11	0.99
Female	95 (86%)	92 (81%)	187 (84%)	0.401
Disease duration (years)	11 (1–42)	10 (0.8–45)	11 (0.8–45)	0.094
DAS28-CRP	5.8 ± 0.8	5.8 ± 0.9	5.8 ± 0.9	0.791
HAQ-DI	1.8 ± 0.6	1.8 ± 0.6	1.8 ± 0.6	0.753
Prednisone treatment	76 (68%)	88 (78%)	165 (74%)	0.11
Prednisone dosage (mg/day)	8 (2–20)	10 (2–15)*	10 (2–20)*	0.243
MTX (mg/week)	14 ± 4	14 ± 4	14 ± 4	0.351
Previous biologic: None/mAb/Eta	8 (7%)/66 (60%)/37(33%)	7 (6%)/70(62%)/36(32%)	15 (7%)/136 (61%)/73(33%)	0.914
Positive RF	76 (69%)	68 (61%)	144 (65%)	0.226
Positive anti-CCP	92 (83%)	78 (70%)	170 (76%)	0.02
Serum IgG level (g/L)	11.8 (4.6–19.2)	12 (5.4–23.3)	12 (4.6–23.3)	0.564
CRP level (mg/L)	12 (0.4–139)	11 (0.3–102)	11 (0.3–139)	0.938
Radiographic erosions	100 (90%)	98 (87%)	198 (88%)	0.432
MTX duration (years)	3.6 (0.1–20.8)	3.2 (0.3–20.5)	3.5 (0.1–20.8)	0.856
Time since last anti-TNF (months)	2.7 (0.6–64.4)	2.8 (1.2–73.6)	2.7 (0.6–73.6)	0.458

Values are shown as means and standard deviations (mean ± SD) or medians (range) for continuous data, and *n* (%) for qualitative data

*Some patients had a deviation protocol concerning the authorized prednisone dosage

*anti-CCP* anti-cyclic citrullinated peptide antibody, *CRP* C-reactive protein, *DAS28* Disease Activity Score in 28 joints, *Eta* etanercept, *HAQ-DI* Health Assessment Questionnaire—Disease Index, *IL* interleukin, *mAb* monoclonal antibody, *MTX* methotrexate, *RF* rheumatoid factor, *TNF* tumor necrosis factor

### Serum IL-33 levels

**Fig. 1:**
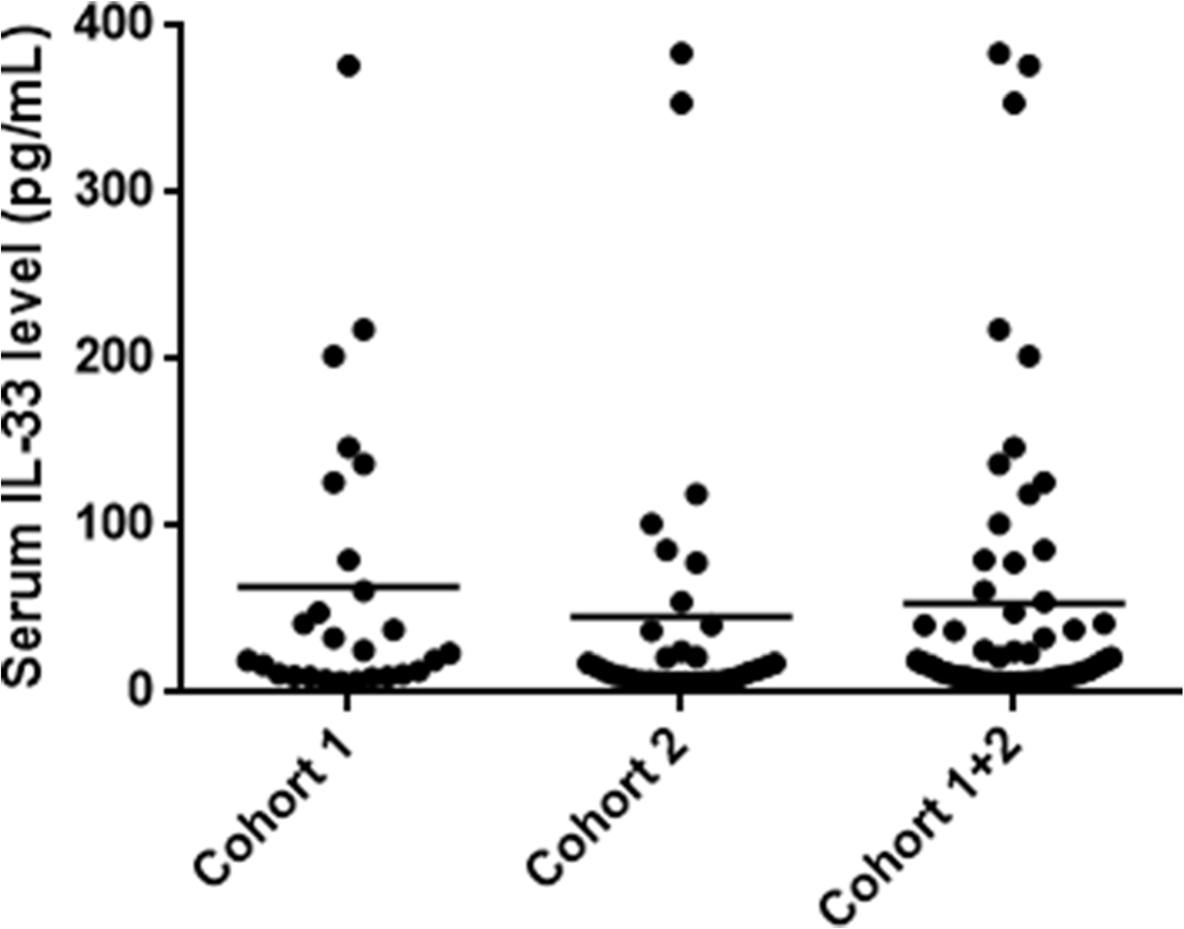
Serum IL-33 levels in patients from cohort 1, cohort 2, and the combined cohorts. Dosage performed with Quantikine ELISA IL-33 kit (R&D Systems). Each dot represents one patient; means are presented. *IL* interleukin

Using the validated Quantikine assay, most of the patients had undetectable serum IL-33: 84/111 (76%) and 39/74 (53%) in cohorts 1 and 2, respectively. In patients with detectable IL-33, mean serum levels were 63.39 ± 87.31 pg/mL and 45.35 ± 87.19 pg/mL in cohorts 1 and 2, respectively (Fig. 1).

### Factors associated with subsequent response to RTX at week 24 in cohort 1

Among the 111 patients, a total of 84 patients (76%) were classified as EULAR responders to RTX at week 24; 27 patients (24%) were non-responders. Detectable serum IL-33 was found in 27 (24.3%) patients. In univariate analysis, the three explanatory variables including DAS28-CRP >5.1, high serum IgG level, and auto-antibody status were associated with RTX response and were included in the multivariate model since they had a *p* value <0.15 for the association with EULAR response (Table 2).

**Table 2:** Factors associated with rituximab response in cohorts 1 and 2

	Characteristic		EULAR non-responders	EULAR responders	Univariate	*p* value	Multivariate	*p* value
					OR (95% CI)		OR (95% CI)	
Cohort 1 (*n* = 111)	DAS28-CRP	3.2–5.1 (Ref)	12 (46.2%)	14 (53.8%)	4.0 (1.55–10.36)	0.004	5.24 (1.85–14.85)	0.002
		>5.1	15 (17.6%)	70 (82.4%)				
	RF or anti-CCP	Negative (Ref)	7 (41.2%)	10 (58.8%)	2.59 (0.88–7.67)	0.08	3.48 (1.04–11.60)	0.04
		Positive	20 (21.3%)	74 (78.7%)				
	IgG > ULN (g/L)	No (Ref)	21 (30.9%)	47 (69.1%)	2.76 (1.01–7.52)	0.048	2.82 (0.97–8.14)	0.056
		Yes	6 (14.0%)	37 (86.0%)				
	Detectable IL-33	No (Ref)	23 (27.4%)	61 (72.6%)	2.17 (0.62–6.95)	0.19	–	–
		Yes	4 (14.8%)	23 (85.2%)				
Cohort 2 (*n* = 74)	Disease activity DAS28-CRP	3.2–5.1 (Ref)	14 (36.8%)	24 (63.2%)	2.92 (0.97–8.73)	0.056	–	–
		>5.1	6 (16.7%)	30 (83.3%)				
	RF or anti-CCP	Negative (Ref)	2 (100%)	0 (0%)	14.72 (0.34–629.71)	0.16	32.76 (0.7–999)	0.076
		Positive	18 (25.0%)	54 (75%)				
	IgG > ULN (g/L)	No (Ref)	15 (37.5%)	25 (62.5%)	2.10 (0.58–7.57)	0.26	–	–
		Yes	4 (22.2%)	14 (77.8%)				
	Detectable IL-33	No (Ref)	14 (35.9%)	25 (64.1%)	2.71 (0.91–8.10)	0.07	3.73 (1.12–12.38)	0.03
		Yes	6 (17.1%)	29 (82.9%)				

Values are given as *n* (%) of responders or non-responders with a given characteristic

Rituximab response was evaluated at week 24 according to EULAR response

*anti-CCP* anti-cyclic citrullinated peptide antibody, *CI* confidence interval, *DAS28-CRP* Disease Activity Score in 28 joints by C-reactive peptide, *EULAR* European League Against Rheumatism, *Ig* immunoglobulin, *OR* odds ratio, *Ref* referent, *RF* rheumatoid factor, *ULN* upper limit of normal (i.e*.*, 12.7 g/L)

The multivariate analysis indicated that high disease activity (OR 5.24, 95% CI 1.85–14.85) and auto-antibody status (OR 3.48, 95% CI 1.04–11.60) were independently associated with RTX response. A non-significant association between EULAR response and high serum IgG level was found (OR 2.82, 95% CI 0.97–8.14) (Table 2). There was no association with serum IL-33 detection in multivariate analysis in cohort 1.

### Factors associated with subsequent response to RTX at week 24 in cohort 2

In order to replicate these results in RA patients from routine care, we tested whether the DAS28-CRP >5.1, high serum IgG level, auto-antibody status, and detectable serum IL-33 were associated with response to RTX in cohort 2 in which there were 73% EULAR responders among 74 patients.

In univariate analysis, high disease activity and detectable serum IL-33 were associated with RTX response, but not auto-antibody status or high serum IgG (Table 2). In multivariate analysis, detectable serum IL-33 was the sole factor associated with RTX response (OR 3.73, 95% CI 1.12–12.38), with a non-significant association for auto-antibody status (OR 32.76 (0.7– 999). The presence of RF and anti-CCP auto-antibodies in 97% of the patients in cohort 2 might limit analysis on these markers.

### Factors associated with subsequent response to RTX at week 24 in the combination of cohorts 1 and 2

**Fig. 2:**
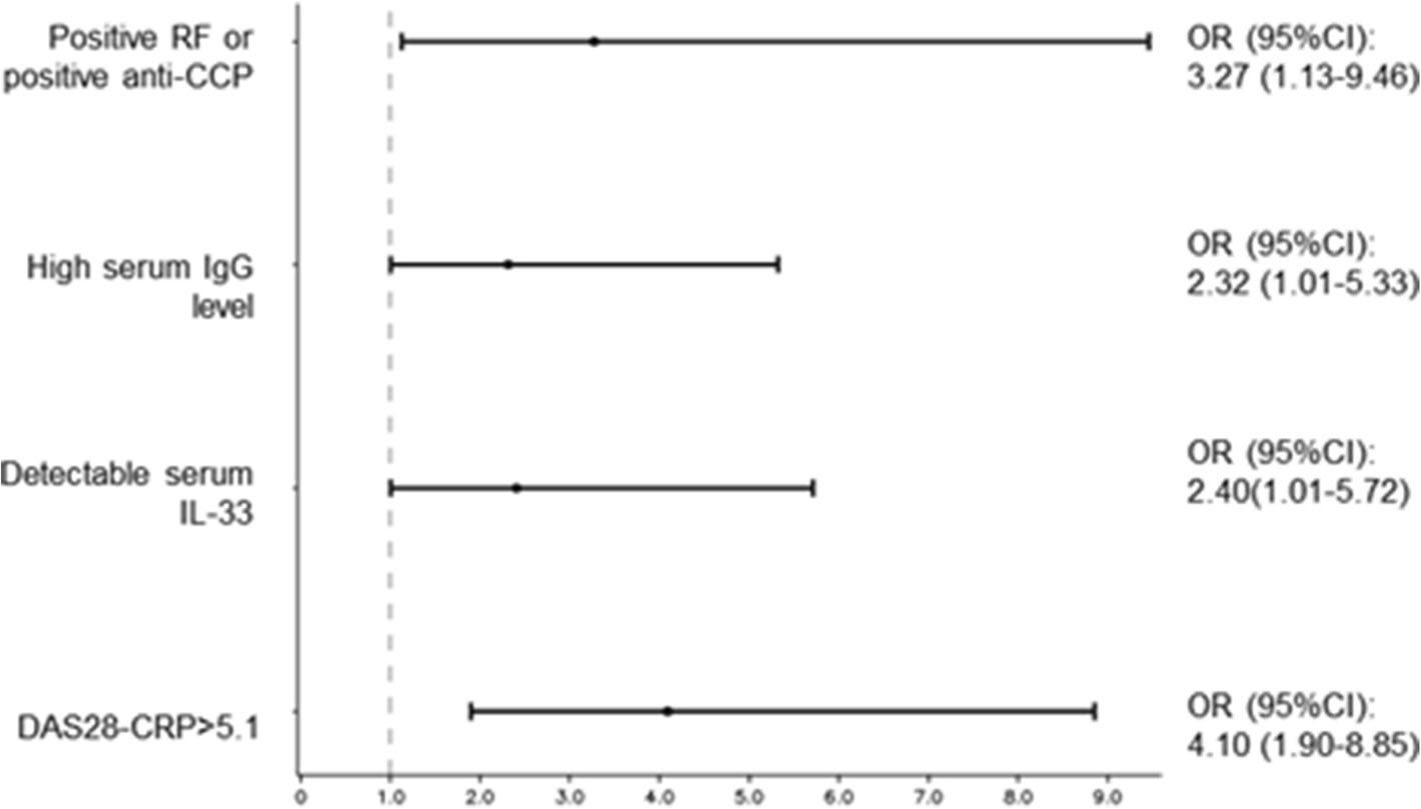
Association between the four explanatory variables and EULAR response at 24 weeks after the first rituximab infusion in the combination of cohorts 1 and 2. Results from the multivariate analysis are presented as odds ratios (*OR*) (95% confidence intervals (*CI*)) for each factor. *anti-CCP* anti-cyclic citrullinated peptide antibody, *DAS28-CRP* Disease Activity Score in 28 joints by C-reactive peptide, *IL* interleukin, *RF* rheumatoid factor

**Fig. 3:**
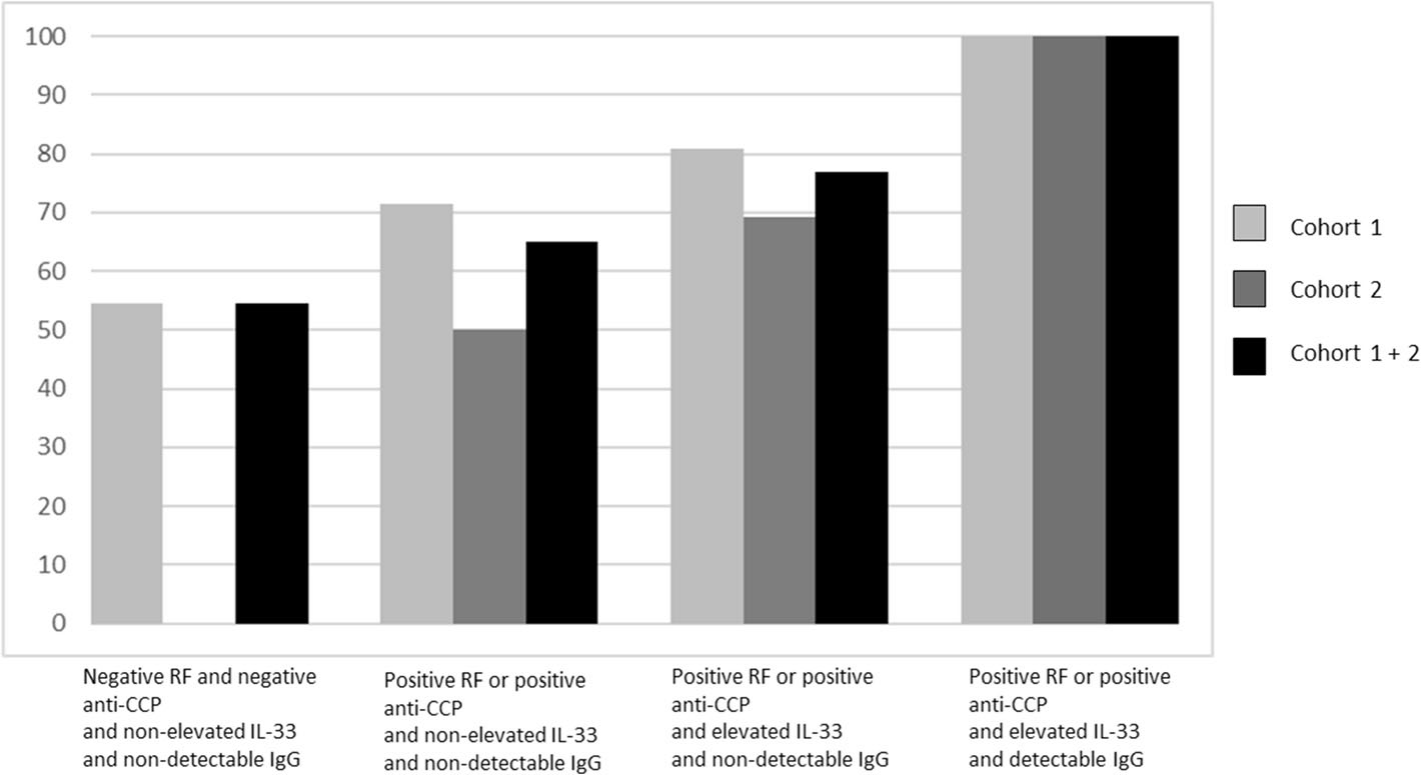
Frequency of EULAR response according to the presence of auto-antibodies, the detectability of serum IL-33, and a serum IgG above the upper limit of normal in cohort 1, cohort 2, and the combined cohorts. Results are presented for patients having auto-antibodies and/or elevated serum IL-33 level and/or elevated serum IgG level compared with patients having no auto-antibodies, a non-elevated serum IL-33 and a non-elevated IgG level (referent). *anti-CCP* anti-cyclic citrullinated peptide antibody, *Ig* immunoglobulin, *IL* interleukin, *RF* rheumatoid factor

Multivariate analyses indicated that four factors were independently and significantly associated with response to RTX: high disease activity (OR 4.10, 95% CI 1.90–8.85), auto-antibody status (OR 3.27, 95% CI 1.13–9.46), high IgG level (OR 2.32, 95% CI 1.01–5.33), and detectable serum IL-33 (OR 2.40, 95% CI 1.01–5.72) (Table 3 and Fig. 2). Since the presence of RF and/or anti-CCP antibodies and high serum IgG level have already been identified as predictive markers of subsequent EULAR response, we aimed to investigate whether the addition of detectable serum IL-33 increased the likelihood of response. While 55% of patients without these three factors responded to RTX, the presence of RF and/or anti-CCP antibodies and elevated serum IgG level further increased the likelihood of this response (77% responders; OR = 2.72, 95% CI 0.67–10.98; *p* = 0.16 versus patients without any of these three factors). When we added detectable serum IL-33 to these two factors, 100% of the patients with the three risk factors (corresponding to 9% of the combined population) responded to RTX (OR 29.61, 95% CI 1.30–674.79; *p* = 0.034 versus patients with none of the three risk factors) (Fig. 3).

**Table 3:** Factors associated with rituximab response in the combination cohort (cohorts 1 and 2)

Characteristics		EULAR non-responders (*n* = 47)	EULAR responders (*n* = 138)	Univariate OR (95% CI)	*p* value	Multivariate OR (95% CI)	*p* value
DAS28-CRP	3.2–5.1 (Ref)	26 (40.6%)	38 (59.4%)	3.26 (1.64–6.47)	0.0007	4.10 (1.90–8.85)	0.0003
	>5.1	21 (17.4%)	100 (82.6%)				
RF or anti-CCP	Negative (Ref)	9 (47.4%)	10 (52.6%)	3.03 (1.15–8.001)	0.025	3.27 (1.13–9.46)	0.03
	Positive	38 (22.9%)	128 (77.1%)				
IgG > ULN (g/L)	No (Ref)	36 (33.3%)	72 (66.7%)	2.55 (1.16–5.60)	0.02	2.32 (1.01–5.33)	0.048
	Yes	10 (16.4%)	51 (83.6%)				
Detectable IL-33	No (Ref)	37 (30.1%)	86 (69.9%)	2.24 (1.03–4.87)	0.04	2.40(1.01–5.72)	0.047
	Yes	10 (16.1%)	52 (83.9%)				

Values are given as *n* (%) of responders or non-responders with a given characteristic

Rituximab response was evaluated at week 24 according to EULAR response

*anti-CCP* anti-cyclic citrullinated peptide antibody, *CI* confidence interval, *DAS28-CRP* Disease Activity Score in 28 joints by C-reactive peptide, *EULAR* European League Against Rheumatism, *Ig* immunoglobulin, *OR* odds ratio, *Ref* referent, *RF* rheumatoid factor, *ULN* upper limit of normal (i.e*.*, 12.7 g/L)

If we restricted the analysis to seropositive patients from both cohorts (94/111 from cohort 1 and 72/74 from cohort 2), IL-33 detection was still highly predictive of EULAR response (OR 3.03, 95% CI 1.24–7.4; *p* = 0.015), confirming that this biomarker was independent of seropositivity.

## Discussion

In this study, we have identified serum IL-33 detection as a novel biomarker associated with RTX response in RA, in addition to auto-antibody status, in real life patients.

This study was based on our previous observation of the upregulation of IL-33 mRNA expression in the whole blood that was associated with RTX response in a microarray study performed in patients randomly selected from the SMART trial [20]. Since the IL-33 protein may be easily quantified in the serum, we first assessed serum IL-33 levels in the same population using the DuoSet ELISA IL-33 kit (R&D System) and preliminary reported in an abstract that the serum IL-33 level was associated with the RTX response [23]. However, additional experiments in patients with Sjögren’s syndrome or RA have raised caution about the accuracy of this kit for sera measurements [24]. Consequently, we discarded these preliminary results and examined serum IL-33 again with an accurate ELISA kit (Quantikine) validated for sera, in two separate and then merged populations.

Here, we have found in univariate and multivariate analysis a significant association between serum IL-33 detection and EULAR response in cohort 2. Furthermore, when we combined the two cohorts, the association was significant with an odds ratio in the same range as high serum IgG level (i.e., approximately 2). We thus confirmed at the protein level the results found at the mRNA level, which demonstrates that transcriptomic analysis with non a-priori hypotheses might open the way to new pathogenic pathways.

This association was independent of strong predictive factors associated with RTX response, especially the presence of RF or anti-CCP antibodies, which strengthens the possible interest in serum IL-33 assessment. We previously reported that patients with a presence of auto-antibodies and high serum IgG level had a better response to RTX in comparison with patients having none of these characteristics [25]. If serum IL-33 detection is added to these two predictive factors, the likelihood of response to RTX reaches 29.6 (95% CI 1.3–675) in comparison with absence of these three factors. Indeed, 100% of patients displaying these three factors simultaneously were responsive to RTX in our combined cohort. However, these patients represented 9% of the study population.

Auto-antibody status was associated with RTX response in SMART, as previously reported [25], but also in the combination of cohorts. The absence of association in cohort 2 alone may be explained by the presence of these auto-antibodies in almost all the patients (97%) limiting analysis on these markers. Lastly, as in every trial, it is easier to have a response when the starting DAS28 is high and high disease activity was associated with a better response to RTX, as previously observed in SMART [25].

Despite a number of strengths, and especially the use of a replication cohort, this study has several limitations. First, serum IL-33 assessment needs the use of an accurate assay such as the Quantikine kit, but the relatively low frequency of patients with detectable IL-33 level (33.5% in the merged population) justifies qualitative IL-33 detection rather than quantitative IL-33 values. Second, we have no data on the association between serum IL-33 detection and response to other biologic agents in RA, limiting our findings to RTX. Third, the association between serum IL-33 detection and response to RTX is statistically significant, but the usefulness of such a measurement for clinical practice needs to be further investigated. Finally, the presence of RF and anti-CCP auto-antibodies in 97% of the patients in cohort 2 might limit analysis on these markers, as well as the difference in terms of high disease activity (DAS 28-CRP >5.1) frequency between the two cohorts (cohort 1 = 77% versus cohort 2 = 49%).

## Conclusions

In conclusion, serum IL-33 assessment with a robust assay may represent a novel, easy biomarker predicting RTX response in synergy with auto-antibody status and high serum IgG level. Such a finding concerning these three biomarkers being easy to monitor in clinical practice represents a further step towards the goal of personalized medicine to determine the best approach to the therapeutic management of RA [27].

## Additional file

Additional file 1: Table S1. Baseline characteristics of RA patients from Clermont and from Leeds. (DOC 33 kb)

## Abbreviations

anti-CCP: Anti-cyclic citrullinated peptide
CI: Confidence interval
CRP: C-reactive protein
DAS28: Disease Activity Score in 28 joints
ELISA: Enzyme-linked immunosorbent assay
ESR: Erythrocyte sedimentation rate
EULAR: European League Against Rheumatism
Ig: Immunoglobulin
IL: Interleukin
MTX: Methotrexate
OR: Odds ratio
RA: Rheumatoid arthritis
RF: Rheumatoid factor
RTX: Rituximab
ST: Suppression of tumorigenicity
TNF: Tumor necrosis factor
ULN: Upper limit of normal
