# Erratum to: Serum IL-33, a new marker predicting response to rituximab in rheumatoid arthritis

**DOI:** 10.1186/s13075-017-1221-4

**Published:** 2017-01-23

**Authors:** Jérémie Sellam, Elodie Rivière, Alice Courties, Paul-Olivier Rouzaire, Barbara Tolusso, Edward M. Vital, Paul Emery, Gianfranco Ferracioli, Martin Soubrier, Bineta Ly, Houria Hendel Chavez, Yassine Taoufik, Maxime Dougados, Xavier Mariette

**Affiliations:** 1 Université Paris 06, AP-HP St-Antoine hospital, Rheumatology Department, INSERM UMRS_938, DHU i2B, Paris, France; 2 Université Paris-Sud, AP-HP Hôpitaux Universitaires Paris-Sud, Rheumatology Department, Center for Immunology of Viral Infections and Autoimmune Diseases INSERM U1184, Le Kremlin Bicêtre, France; 3 Biological Immunology Department, ERTICa Research Group, Clermont-Ferrand University Hospital, Clermont-Ferrand, EA4677 France; 4 Rheumatology Department, Catholic University of the Sacred Heart, Roma, Italy; 5 NIHR Leeds Musculoskeletal Biomedical Research Unit, Leeds Teaching Hospitals NHS Trust, Leeds, UK and Leeds Institute of Rheumatic and Musculoskeletal Medicine, University of Leeds, Leeds, UK; 6 Rheumatology Department, Clermont-Ferrand University Hospital, Clermont-Ferrand, France; 7 AP-HP Bicêtre Hospital, Biological Immunology Department, INSERM U1184, Le Kremlin Bicêtre, France; 8 Department of Rheumatology - Hôpital Cochin, Paris Descartes University, Assistance Publique - Hôpitaux de Paris, INSERM (U1153), Clinical Epidemiology and Biostatistics, PRES Sorbonne Paris-Cité, Paris, France; 9 Service de Rhumatologie, Hôpital de Bicêtre, 78 rue du Général Leclerc, Le Kremlin Bicêtre, 94275 France; 10 Service de Rhumatologie, Hôpital Saint-Antoine, 184 rue du Faubourg Saint-Antoine, Paris, 75012 France

## Erratum

Unfortunately, after publication of this article [1], it was noticed that the name of Gianfranco Ferracioli was incorrectly spelled as Gianfranco Ferraciolli. The corrected author list can be seen above and the original article has been updated to correct this error.
