# Supplementary material for: Serum IL-33, a new marker predicting response to rituximab in rheumatoid arthritis
Source: Arthritis Res Ther. 2016 Dec 13;18:294. doi: 10.1186/s13075-016-1190-z (PMC5154136; doi:10.1186/s13075-016-1190-z)
Supplement: Additional file 1: Table S1. — Baseline characteristics of RA patients from Clermont and from Leeds. (DOC 33 kb) [file 13075_2016_1190_MOESM1_ESM.doc]

Additional file

|  | Patients from Clermont  N=42 | Patients from Leeds  N = 32 |
| --- | --- | --- |
| Age (years) | 60 ± 12 | 58 ± 14 |
| Disease duration (years) | 10 (0-53) | NA |
| DAS28-CRP | 4.9 ± 0.9 | 5.4 ± 1.3 |
| Prednisone treatment (number of patient and %) | 24 (57%) | NA |
| Prednisone dosage | 7.5 (3-17.5) | NA |
| MTX (mg/week) | 15.6 ± 3.4 | NA |
| Positive RF | 35 (85%), ND =1 | 22 (73%), ND = 2 |
| Positive anti-CCP | 37 (88%) | 32 (100%) |
| Serum IgG level (g/L) | 10.4 (5.6-22.8) | 12.7 (5.6-19.5) |
| CRP level (mg/L) | 9.8 (0.6-92) | NA |

**Table S1**: Baseline characteristics of RA patients from Clermont and from Leeds.

RF: rheumatoid factor; anti-CCP:anti-citrullinated cyclic peptide antibodies;DAS28: Disease Activity Score 28; HAQ-DI: Health Assessment Questionnaire – Disease Index CRP: C reactive protein; MTX: methotrexate; mAb: monoclonal antibody; eta: etanercept.

NA : not available

Reported values are for continuous data: means and standard deviations (mean ± SD) or medians (extremes), for qualitative data: number of patients (n) and frequency (%)
